# „Man lasse sich ja nicht auf Diskussionen über ‚Samenverluste‘ ein“: Das wilde Leben des Urologen Felix Schlagintweit (1868–1950)

**DOI:** 10.1007/s00120-023-02106-4

**Published:** 2023-06-15

**Authors:** Florian G. Mildenberger, Friedrich H. Moll

**Affiliations:** 1 Institut für Geschichte, Theorie und Ethik der Medizin, Heinrich-Heine-Universität Düsseldorf, Düsseldorf, Deutschland; 2 Curator Museum, Bibliothek und Archiv zur Geschichte der Urologie, Deutsche Gesellschaft für Urologie e. V., Düsseldorf-Berlin, Düsseldorf und Berlin, Deutschland; 3 Urologische Klinik, Kliniken der Stadt Köln GmbH, Betriebsteile Holweide und Merheim, Neufelder Str. 32, 51067 Köln, Deutschland

**Keywords:** Urologie, Wissenschaftstheorie, Diagnostische Instrumente, Sanatorium, Bad Brückenau, Nationalsozialismus, Urology, Theory of science, Cystoscope, Sanatorium, Bad Brueckenau, National socialism

## Abstract

Felix Schlagintweit war Klinikassistent, Sanatoriumsbetreiber, Schriftsteller und Urologe in freier Praxis. Er verbesserte die diagnostischen Methoden des Faches (Zystoskop) wesentlich und interessierte sich für die Psychoanalyse. Einseitig operativen Therapien erteilte er ebenso eine Absage wie der alleinigen Konzentration auf die Somatik. Seiner Ansicht nach waren konservative Therapieoptionen oftmals mindestens ebenso wirksam. Da er sich auch politisch nicht anpassen wollte, wurde er spätestens nach 1933 aus dem fachinternen Diskurs getilgt und erst spät wieder entdeckt.

## Abstract (en)

Felix Schlagintweit worked in a medical clinic, was co-owner of a sanatorium, had a private practice and wrote fictional books. He massively improved diagnostic methods (e.g., cystoscope) and was interested in psychoanalysis. He rejected the effectiveness of surgical treatment alone and also sole use of psychosomatics. In his view, conservative treatment options were often at least as effective. Because Schlagintweit refused to take part in national socialism, he was purged from professional discourse after 1933 and was only later were his contributions to the history of urology rediscovered.

## Einleitung

Gerät eine bestimmte Form der Therapie oder der Diagnostik außer Gebrauch, so werden alsbald auch die bedeutendsten Vertreter der nun als zu vernachlässigend eingestuften Form der eigenen Arbeit aus dem jeweils aktuellen Fachdiskurs gedrängt. Zunächst werden die Gelehrten, mögen sie noch so bekannt gewesen sein, nicht mehr auf Tagungen eingeladen, so dass jüngere Fachvertreter sie und ihre Ideen nicht mehr kennenlernen können. Es folgt der allmähliche Ausschluss aus den Fachzeitschriften und weiteren Netzwerken und darauf folgt sukzessive das Vergessen. Einige wenige der Betroffenen entgehen der endgültigen Nemesis durch Abfassung von Memoiren, die zumeist darauf angelegt sind, versöhnlich zu erscheinen, so als ob der erzwungene Rückzug der letzten Jahre eine persönliche Entscheidung gewesen sei. Doch einer hielt sich nicht einmal daran.

Felix Schlagintweit blieb bis zuletzt ein streitbarer Zeitgenosse, der kein Blatt vor den Mund nahm [1]. Die Folge war, dass seine launige Autobiografie zwar mehrfach aufgelegt wurde und besonders im Süddeutschland der Nachkriegszeit bei Urologen eine weite Verbreitung fand, wie uns Zeitzeugen mehrfach berichteten. Auch eine große Zahl an Lesern außerhalb der Urologie bzw. der Medizin fand das Werk; der Name aber wurde zügig aus der Erinnerung der Fachgeschichte getilgt.

## Ein großer Sohn aus einer kleinen Nebenlinie

Als in der zweiten Hälfte des 19. Jahrhunderts das Zeitalter des Kolonialismus und der Begeisterung für fremde Länder und deren wissenschaftliche und militärische Erforschung auch die deutschen Kleinstaaten erreichte, besaß das Königreich Bayern bereits ein Trio an nationalen Helden, die „Gebrüder Schlagintweit“: die geadelten Brüder Hermann (1826–1882) und Robert (1833–1885) sowie den bei Kaschgar ermordeten Adolf (1829–1857) [2].

Sie alle drei waren die Söhne des Augenarztes Joseph Schlagintweit (1792–1854). Nicht ganz so bedeutsam war sein Neffe, der Bezirksingenieur Anton Schlagintweit (1835–1904) aus Regensburg, der jedoch seinen beiden Söhnen Felix (1868–1950) und Oskar (1875–1964) das Medizinstudium ermöglichte und sie bereits als Schüler durch die berühmten Verwandten in die Münchner Gesellschaft einführen ließ. Oskar studierte nach der Reifeprüfung 1895 in München und Jena Medizin und bestand 1901 das medizinische Staatsexamen und promovierte.

## Kurze Vita Felix Schlagintweit

Der am 21. September 1868 in Bamberg geborene Felix Schlagintweit verbrachte seine frühe Kindheit auf einer kleinen Bahnstation im niederbayerischen Geiselhöring, Landkreis Straubing. Nach einem vorübergehenden Aufenthalt in Regensburg kehrt die Familie nach Bamberg zurück, wo sie schon zuvor gelebt hatte.

Schlagintweit besuchte in Bamberg das Gymnasium mit einem einjährigen Zwischenaufenthalt im Jahre 1886 am Internat in Burghausen. Wieder zurück in Bamberg begeistert er sich für Musik und spielt am Kontrabass im Schulorchester mit. Während der Gymnasialzeit las er nach eigenen Angaben Heinrich Heine, Emile Zola und Paul Heyse, dessen Roman „Im Paradiese“ ihn „auf das Münchner Künstlerleben vorbereitet“ [3].

Felix studierte 1889 bis 1894 Medizin an der Ludwigs-Maximilian-Universität in München und in Erlangen, promovierte in Erlangen im Jahre 1893 [4] und erhielt danach im Jahre 1894 seine Approbation.

In den folgenden beiden Jahren verdingte er sich als Volontärassistent an der Medizinischen Klinik der Universität, deren Direktor Hugo v. Ziemssen (1829–1902) sein Talent erkannte und ihm nahelegte, sich auf eine frei gewordene Stelle als Spezialarzt für Harnkranke in Bad Brückenau zu bewerben. An Ziemssens Klinik war er mit den neuesten Trends aus physikalischen Heilweisen und Elektrotherapie vertraut gemacht worden [5, 6 S. 7, 7]. 1901 absolvierte er seine spezialärztliche Ausbildung in Paris bei Felix Guyon (1831–1920) und Joaquin Albarran (1860–1912), ehe er sich mit seinem Bruder zusammentat, um gemeinsam in Bad Brückenau zu arbeiten. In seinen Memoiren schilderte Felix Schlagintweit anschaulich die unbeholfene Rezeption neuester hygienischer Erkenntnisse in der praktischen Medizin am Beispiel des städtischen Krankenhauses in Bamberg, wo er bei Lothar Nebinger (1846–1894) ein Praktikum absolviert hatte:

„Ich kam gerade in die antiseptische Zeit der Chirurgie. Karbolsäure floß in Strömen und wurde sogar in den Operationssälen als Karbolspray über uns alle vernebelt. Das ganze Krankenhaus roch überlaut nach Jodoform, wie lange noch die kleinen veralteten Krankenhäuser in der Provinz. Nebinger schüttete es eßlöffelweise in die offene, eitrige Bauchhöhle. Das Verbandszeug wurde unsterilisiert einer alten, wunderbaren, barocken Kommode aus der Fürstbischofszeit entnommen, die mitten im Operationssaal glänzte.“ [3, S. 68]

Ihm schwebte eine Synthese aus Hygiene, modernisierter Diagnostik und praktischer Therapie vor, doch fanden er und sein Bruder mit ihren Überlegungen in der *Scientific Community* der operativen Medizin, der Venerodermatologen und Balneologen anfangs nur wenig Zuspruch. Infolgedessen beschloss Felix Schlagintweit die urologischen Arbeitstechniken zu verbessern, während der Bruder Oskar Möglichkeiten zur Gründung einer Privatklinik auslotete.

## Bad Brückenau

Auf diesen Gedanken waren die Brüder 1902 bei der gemeinsamen Arbeit in Bad Brückenau gekommen. Das hierfür nötige Geld dürfte Felix Schlagintweits Ehefrau Emilie Nathan (1872–1938) beigesteuert haben, die aus einer Bamberger Brauereidynastie stammte und die Felix 1895 ehelichte.^1^ Brückenau war damals die „kleine Schwester“ des mondänen Staatsbades Bad Kissingen und von diesem 5 Wegstunden entfernt [8, S. 67]. Ein „Stahlbad“, ein Moorbad und ein Solebad ermöglichten den Kurbetrieb. Bad Brückenau besaß einen etwas eigentümlichen Ruf als der Ort, an dem König Ludwig I (1786–1868, reg. 1825–1848) mit seiner Mätresse Lola Montez (1821–1861) glückliche Tage verlebte und sich dabei nebenbei selbst um den Thron brachte. Der frühere Fürstenbau diente mittlerweile als Hotel. Mindestens ebenso bekannt wie als Ort für außereheliche Vergnügungen war die Wernarzer Heilquelle für Nieren- und Blasenkranke, deren Wasser abgefüllt und im ganzen Deutschen Reich konsumiert wurde. Die Bad Brückenauer Quellen wurden bereits 1908, nach Einführung eines ersten bayerischen Wassergesetzes [9], als Heilquellen staatlich anerkannt und durch Ausweisung eines umfangreichen Heilquellenschutzgebiets geschützt. Die Quelle war 1749 als zweite Heilquelle Bad Brückenaus entdeckt und gefasst worden und begründete den Ruf Bad Brückenaus als Nierenheilbad und gilt als einer der ältesten Versandbrunnen Deutschlands und besaß in der Urologie einen guten Ruf [10, 11, S. 55–58, insbesondere S. 55].

Im Bereich der aufstrebenden Urologie gehörten die Badeärzte in Brückenau, Wildungen oder Karlsbad zu einer wichtigen konstituierenden Gruppe. Damit diese am Urologenkongress teilnehmen konnten, wurde dieser häufig erst nach Ende der Kursaison „*Ende September*“ „*vor Semesterbeginn*“, was den an Hochschulen Tätigen geschuldet war, terminiert.

**Figure 1:**
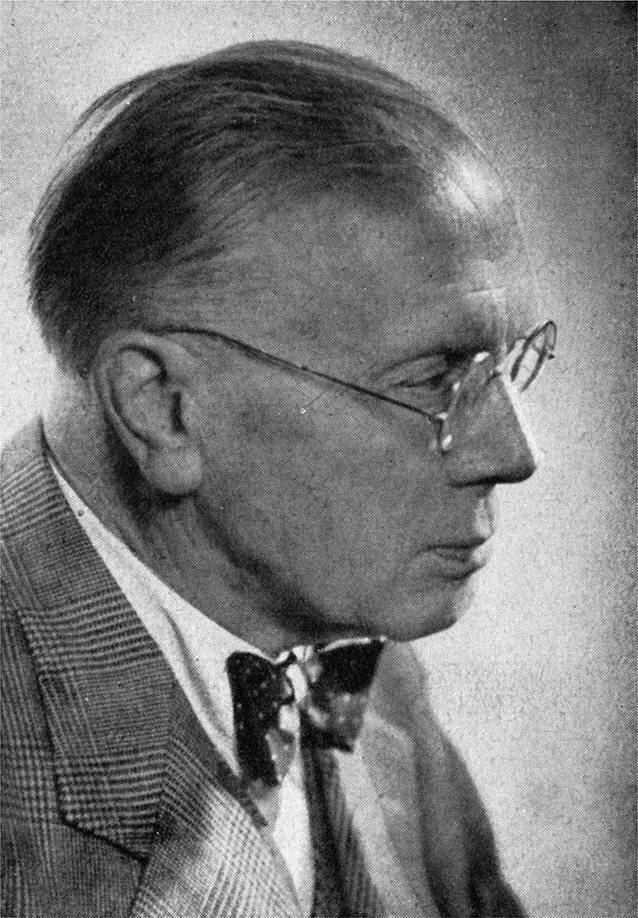

**Figure 2:**
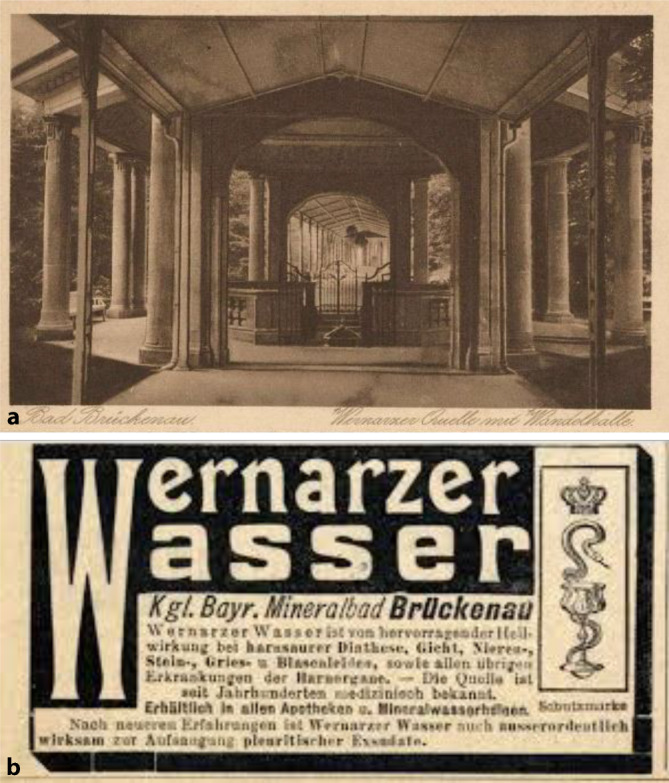

**Figure 3:**
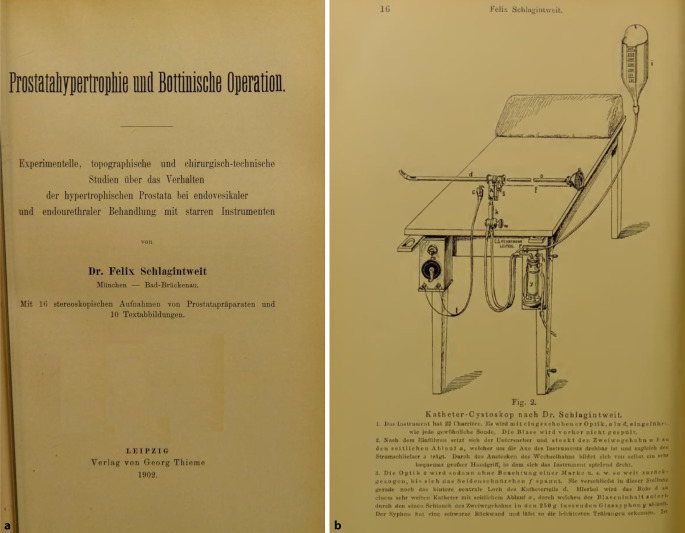

**Figure 4:**
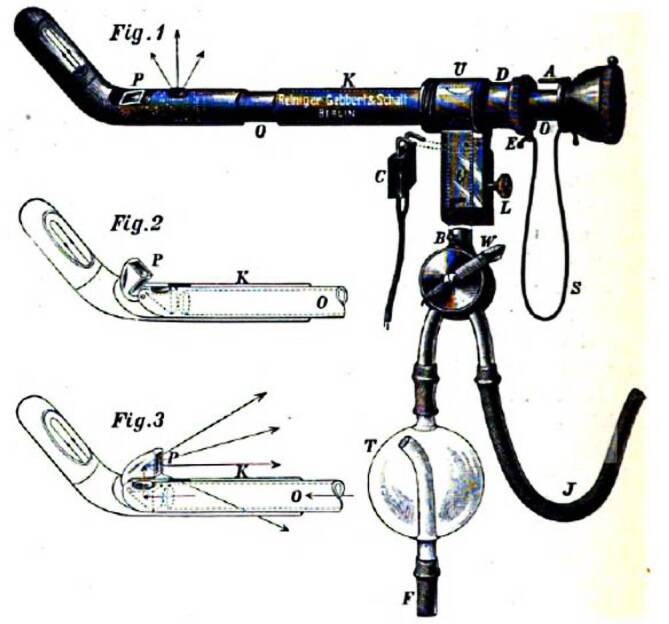

**Figure 5:**
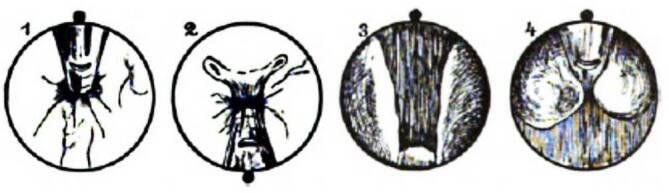

**Figure 6:**
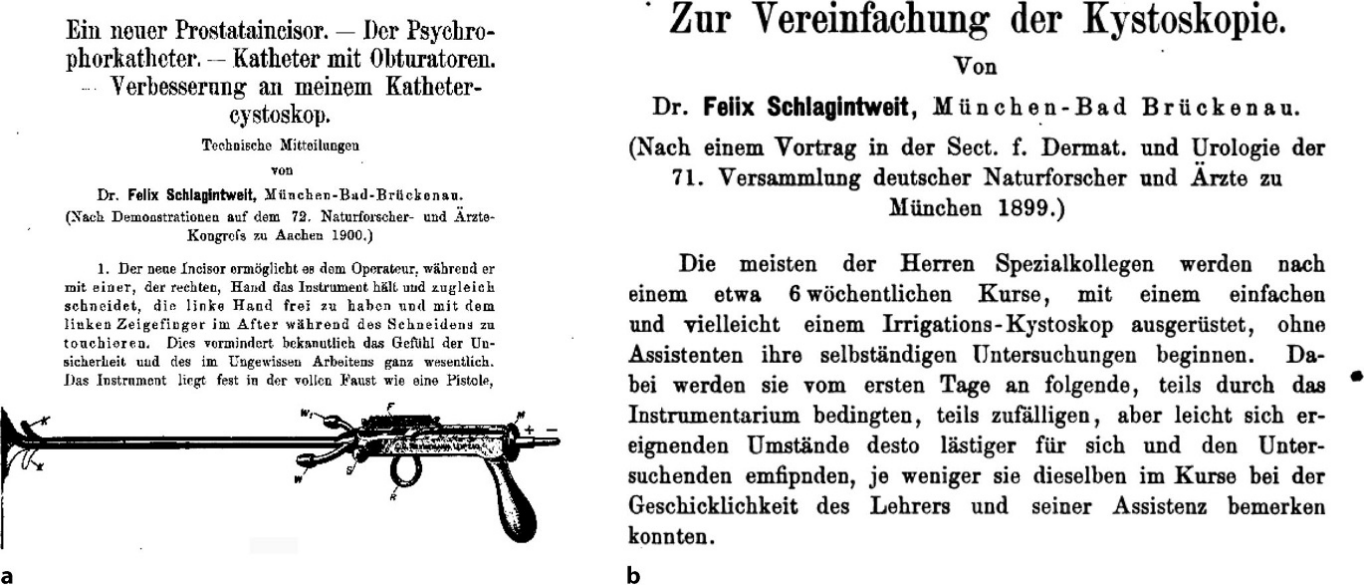

Schlagintweit hatte zur Wernarzer Quelle bereits in einer seiner ersten wissenschaftlichen Publikationen, noch vor der Anerkennung als Staatsbad, ausführlich Stellung genommen [12, 13]. 1898 war er behandelnder Arzt der kurz vor ihrem Tode zur Kur in Bad Brückenau weilenden Kaiserin Elisabeth von Österreich (1837–1898), was die sozial arrivierte Stellung schon des jungen Arztes unterstreicht.^2^ In seinem Nachruf unterstreich sein Münchener Antipode Ludwig Kielleuthner, dass dies ihm eine beachtlichen Patientenzustrom sicherte (Abb. 1, 2, 3, 4, 5, 6) [14].

## Das Rückblickzystoskop nach F. Schlagintweit

Neben einem differenzierten wissenschaftlichen Oevre war Felix Schlagintweit kurz nach der Wende zum 20. Jahrhundert auch an der Weiterentwicklung des Nitzeschen Zystoskops nicht unwesentlich beteiligt.

Zu diesem Zeitpunkt der Zystoskopentwicklung war der Blickwinkel der Optiken noch deutlich eingeschränkt. Dies ermöglichte de facto keinen retrograden Blick auf die Blasenvorderwand oder den Blasenauslass.

Zur Schleimhautbeurteilung, insbesondere bei einer Mittellappenhypertrophie der Prostata oder bei der Suche nach Steinresten nach Lithotripsie, war und ist diese Blickachse aber von entscheidender Bedeutung.

Im Jahre 1903 veröffentlichte Felix Schlagintweit im „Nitze-Oberländerschen Centralblatt“ – nach Ankündigung im Jahre 1901 – seine eigene Zystoskopmodifikation, die er in Zusammenarbeit mit der innovativen Instrumentenbaufirma Reiniger, Gebbert und Schall (heute Siemens Medizintechnik, Erlangen – Berlin) entwickelt hatte. Er selber gab im Jahre 1901 an, dass er bereits seit dem Jahre 1900 an dieser Modifikation arbeite. Schon vorher hatte er ein Spülkatheterzystoskop (der Zystoskoptyp, auf den alle heutigen zurückgehen) publiziert und auf der Naturforscherversammlung vorgestellt, der gegenüber dem von Max Nitze angegebenen wesentliche Vorteile besaß. Schon seine Vorfahren, die „Gebrüder Schlagintweit“, hatten auf dieser bedeutenden Wissenschaftlerzusammenkunft wichtige Ergebnisse vorgestellt.

Für Schlaginweit war das von Maximilian Nitze angegebene Zystoskop III, das einen eingeschränkten rückwärtsgewandten Blick zuließ, zu diesem Zwecke nicht ausreichend gewesen. Er fügt neben einer Instrumentenabbildung auch Skizzen zur neuen Blickrichtung bei, die die Überlegenheit des Instruments einem im Umgang mit dem Instrument Erfahrenen sofort erkennen lassen [15].

Sogar Max Nitze, der publizierten Modifikationen seines eigenen Instruments in der Regel prinzipiell ablehnend gegenüberstand, konnte diese Modifikation durchaus akzeptieren, was die Zeitgenossen wohlwollend aufnahmen.

„Seine unbezweifelbare Fertigkeit in der Anwendung eines optisch noch recht unvollkommenen Hilfsmittels ließ ihn die meisten von anderer Seite kommenden Verbesserungsvorschläge ablehnen, und ich erinnere mich, nur einmal seine rückhaltlose Zustimmung zu einer solchen Neuerung gefunden zu haben. Er widmete sie dem von F. SCHLAGINTWEIT (gesperrt Ringleb) geistreich geplanten Gedanken des ersten Rückblickrohrs, suchte aber das gleiche Ziel auf einem Wege zu erreichen, der größere Gewähr gegen Störungen durch mangelhafte Ausführung bot.“ [16, S. 30]

stellte Otto Ringleb (1875–1946) hierzu fest.

Auch Eugen Joseph (1879–1933), Repräsentant der Berliner Urologie an der ersten Chirurgischen Klinik Ziegelstraße, Berlin, besprach das Schlagintweit-Instrument in seinem Lehrbuch ausführlich (Abb. 4, 5, 6a, b) [17, S. 25].

## Wegbereiter eines neuen medizinischen Spezialfachs

Neben der Beschäftigung mit der Zystoskopie begann Felix Schlagintweit mit Röntgenstrahlen zur Therapie der Prostatahypertrophie zu experimentieren [18]. Doch bereits 1907 thematisierte er – weit früher als die Kollegenschaft – die Gefahr von „Röntgenverbrennung“, weshalb er auf weitere Anwendungen verzichtete [18]. Hier nahm auch Schlagintweits Kritik an neuen Behandlungsmethoden und dem Fortschrittsoptimismus seiner Zeit ihren Anfang. Er wandte sich nun eher konservativen therapeutischen Methoden zu und konzentrierte sich auf die Weiterentwicklung der urologischen Diagnostik. Hierzu gehörte die Entwicklung und Perfektionierung seines 1904 präsentierten „Apparates zur Gefrierpunktbestimmung des Harnes“ [19].

Aufgrund seiner familiären Beziehungen, eigener künstlerischer Begabung und seines gewinnenden Wesens gelang es ihm, Zugang zu Künstlerkreisen zu erlangen (s. unten). Hier war Spiritismus zu dieser Zeit eine angesagte Unterhaltungsform und so nahm auch Schlagintweit an entsprechenden Seancen teil. Im Fall der durch den Parapsychologen und Arzt Albert v. Schrenck-Notzing (1862–1929) als Medium präsentierten „Schlaftänzerin Magdeleine G.“ notierte er kritisch, hier habe wohl eher das Medium die Kontrolle und nicht der Arzt. Der Tanz der leicht bekleideten Dame sagte ihm immerhin zu [20]. Schrenck-Notzing lud ihn nie wieder ein.

Schlagintweit betätigte sich als frühes Mitglied der Deutschen Gesellschaft für Urologie auch standespolitisch und war bis zu Anfang der 1930er-Jahre Gast auf vielen Kongressen der alten DGfU.^3^ Als 1910 der niedergelassene Urologe Constantin Wiedmann seitens des Dermatologen und außerordentlichen Universitätsprofessors Albrecht Notthafft v. Weißenstein (1868–1950) der Kurpfuscherei bezichtigt wurde [21] sprang Schlagintweit dem bedrohten Kollegen bei und half ihm, den Verdacht auszuräumen.

## Versuch einer Habilitation

Schließlich strebte Felix Schlagintweit nach der höchsten Stufe aller akademischen Würden: eine Habilitation im sich gerade formierenden Fach der Urologie. Am 12. Februar 1912 wandte er sich an die hohe medizinische Fakultät der Ludwig Maximilians Universität München mit einem entsprechenden Gesuch. Titel der Habilitationsschrift war „Technik der Diagnose, Operation und Harnleiterbehandlung bei Nierentuberkulose“. Um den zu erwartenden Widerständen der mächtigen chirurgischen Fraktion in der Münchener Medizinischen Fakultät zu begegnen, bezeichnete er die Urologie als

„nicht zu separierendes Nebenfach der Chirurgie“.^4^

Dadurch entwertete er aber seinen eigenen Antrag, denn eine Lehrbefugnis in einem neuen Fach wie der Urologie setzte einen Sondercharakter der Fachrichtung voraus. Da spielte es keine Rolle, dass Felix Schlagintweit versichern konnte, bereits Räumlichkeiten für seine Lehrveranstaltungen organisiert zu haben^5^. Die fakultätsinternen Debatten führten schließlich dazu, dass Schlagintweit sein Habilitationsgesuch am 10. Juni 1912 zurückzog und wenige Tage später seine Unterlagen wiedererhielt. Damit war dieser Versuch einer Habilitation im Fach Urologie an einer Universität im deutschen Reich im Gegensatz zur k. u. k-Monarchie gescheitert. In München sollte dies dann interessanterweise Ludwig Kielleuthner, dem 8 Jahre jüngeren (1876–1972) im gleichen Jahre 1912 gelingen.^6^ Es konnte bisher anhand der Akten nicht genau geklärt werden, ob fakultätsintern zwischen beiden Bewerbern irgendwelche Interferenzen aufgetreten waren oder Kielleuthners Netzwerke effektiver waren. Kielleuthner war sicherlich akademisch etwas breiter aufgestellt. Nach dem Staatsexamen arbeitete er 3 Jahre lang in Wien: am Pathologischen Institut bei Anton Weichselbaum (1845–1920), im Labor bei Karl Landsteiner und als Operationszögling an der II. Chirurgischen Universitätsklinik bei Julius Hochenegg (1859–1940) und am Rothschild-Spital bei Otto Zuckerkandl (1861–1920). Anschließend verbrachte er ein Jahr als Austauscharzt in Paris wie Schlagintgweit bei Felix Guyon und Joaquín Albarrán am Hopital Necker, zuletzt noch für kurze Zeit bei Peter Freyer (1851–1921) in London. Mit dieser Ausbildung war er der operativen chirurgischen Richtung des Fachgebiets zuzurechnen. Auch sein Bewerbungsschreiben ist deutlich akademischer verfasst, was zwischen den Zeilen zu erkennen geben kann, dass die Universität lieber einem rein akademisch tätigen Urologen mit differenzierten Publikationen den Vorrang für die Lehre des „neuen Spezialfaches“ gegeben hat als einen bereits in guten pekuniären Verhältnissen agierenden Badearzt und Endoskopiker mit Praxisklinik an bestem Standort in München, der gleichzeitig kulturelle Interessen als Schriftsteller und Musiker pflegt.^7^

**Figure 7:**
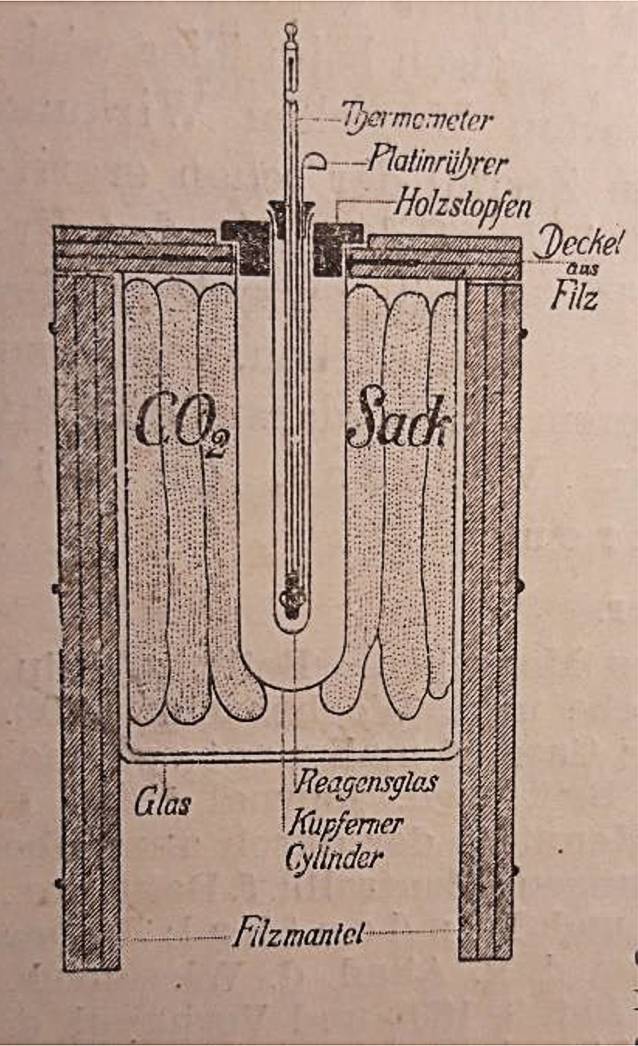

**Figure 8:**
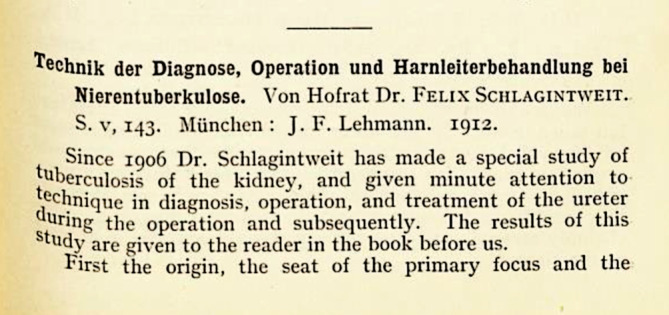

**Figure 9:**
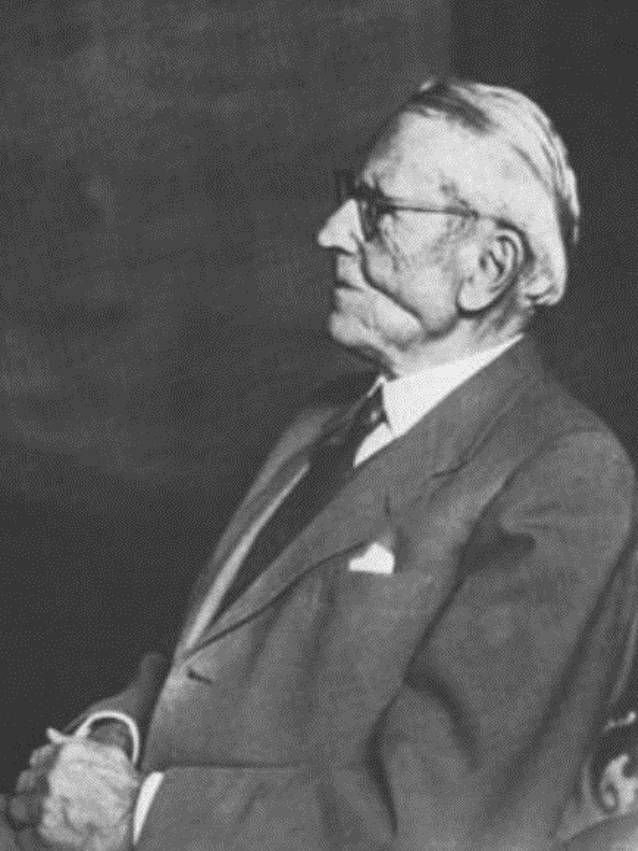

Die zur Habilitation vorgesehene Arbeit Schlagintweits wurde im internationalen Schrifttum positiv besprochen (Abb. 7, 8, 9).

Schlagintweit ließ sich von dem Rückschlag nicht entmutigen und setzte seine Forschungen zur Verbesserung der urologischen Technik fort. Die zurückgezogene Habilitationsschrift publizierte er umgehend in Buchform im Verlag J. F. Lehmanns in München^8^ und ließ darin durchblicken, dass operative und konservative Behandlungsmethoden langfristig vergleichbare Ergebnisse erzielten [22, S. 129f.]. Sollte er dies so in der ursprünglichen, jedoch nicht überlieferten Habilitationsschrift formuliert haben, könnte dies die Chirurgen in der Fakultät nachhaltig verärgert haben.

**Figure 10:**
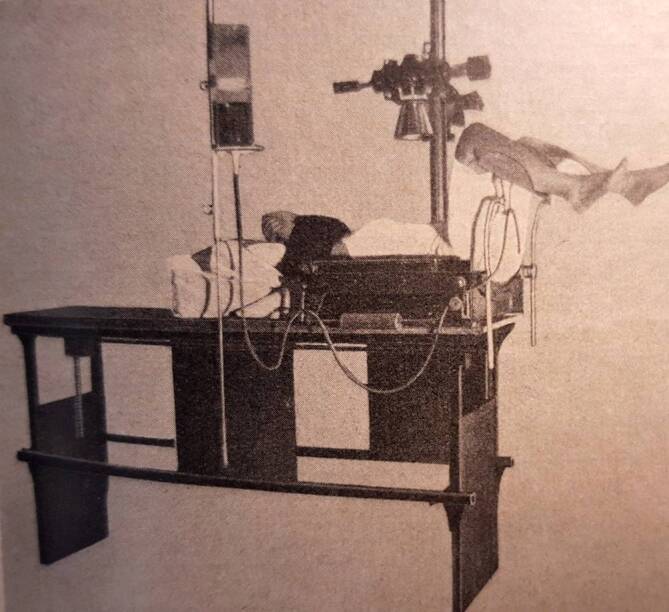

**Figure 11:**
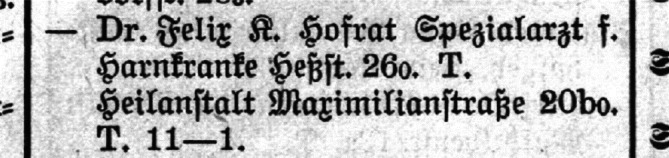

Auch entwickelte er einen eigenen, auf die spezifischen Erfordernisse der Urologie ausgerichteten Röntgentisch und ließ diesen unter seiner Aufsicht ab 1932 produzieren [23] (Abb. 10, 11).

Im Ersten Weltkrieg arbeitete er als Lazarettarzt im Münchner Rot-Kreuz-Krankenhaus und lernte hier am OP-Tisch 1917 die 27 Jahre jüngere Krankenschwester Centa „Monna“ Glier (1895–1961) kennen, die er zur Sekretärin und Geliebten machte. Erst nach dem Tod seiner scheidungsunwilligen Ehefrau erfolgte im Dezember 1938 die Heirat. Beide Ehen blieben kinderlos.

Felix Schlagintweit wurde 1912 mit dem „Titel“ des Hofrates^9^ – vielleicht aufgrund gescheiteter Habilitation an der LMU – geehrt und war ein geachtetes Mitglied der Münchner Gesellschaft [6, S. 15]. Aber bereits zu diesem Zeitpunkt begann die Entfremdung von der urologischen *scientific community*, deren Angehörige eher auf das Skalpell als auf konservative Behandlungsmethoden setzten. Bisweilen erschienen ihm psychotherapeutische Ansichten sinnvoll. In seinen Memoiren notierte er, dass die „psychoanalytische Kunst“ von Nutzen sein konnte [3, S. 141]. Felix Schlagintweit konnte weder in der Klinik noch in der Privatpraxis seine Ideen umsetzen. Hierfür benötigte er so etwas, wie einen eigenen „Zauberberg“. Diesen hatte er sich parallel zu seiner Münchner Karriere sukzessive in der fränkischen Provinz erarbeitet.

## Statt Universität München lieber Bad Brückenau

Wie bereits erwähnt, arbeiteten Oskar und Felix Schlagintweit ab 1901/1902 in der sommerlichen Kursaison in Bad Brückenau. Anfangs praktizierten sie in einem kleinen Haus, in dem (angeblich) Lola Montez (1821–1861), die Geliebte König Ludwigs I. von Bayern (1786–1868), der sie 1847 zur Gräfin Marie von Landsfeld erhob, Quartier bezogen hatte [3, S. 121]. Spätestens 1905 verlegten sie die um einige Belegbetten erweiterte Praxis in das Kurmittelhaus, das sie allmählich zu einer Privatklinik ausbauten.

Für die Jahre 1905 bis 1909 legten die Brüder Schlagintweit einen Rechenschaftsbericht in Buchform ab, der zugleich Einblicke in die Welten der Patienten gewährt. In den 5 Jahren waren nur 25 Patienten mit akuter Urethralgonorrhö aufgenommen worden, aber 233 chronische Fälle [24, S. 11]. Offenbar suchten die Betroffenen erst dann ärztlichen Rat, als die Schmerzen oder Komplikationen unerträglich wurden. 41 Männer wandten sich an das Sanatorium wegen nervöser Störungen, wie sie es selbst nannten. Die Schlagintweits arbeiteten in Gesprächen heraus, dass es sich hierbei um Schuldgefühle und Ängste aufgrund von Masturbation handelte. Solchen Patienten riet man zu möglichst viel Geschlechtsverkehr in ehelicher Verbindung. Die Prostatahypertrophie war ein häufiges Leiden, das 360 Patienten betraf, die zumeist völlig unwissend waren. Daneben machten diejenigen Akteure Sorgen, die sich mit Hilfe von frei verfügbaren Informationen selbst behandelten.

„… gibt es eine ganze Reihe von Patienten, die sich Jahrzehnte lang in der schlampigsten Weise selbst katheterisieren ohne eine Cystitis zu bekommen. Einer benützte 4 Jahre lang seinen Speichel als Kathetergleitmittel und hatte klaren Urin.“ [24, S. 30]

Die allermeisten Ratsuchenden litten jedoch an einer Nephritis (1129 Fälle). Die Grunderkrankung war meist die Gicht, weshalb die Schlagintweits nicht umhin kamen, den zumeist unwilligen Herren eine Lebens- und Ernährungsumstellung nahe zu legen. Vor Ort griff das Brüderpaar zu recht drastischen Therapien:

„… besonders aber Bergabspringen wurden den periodisch an Koliken und Steinabgängen Leidenden empfohlen, solange sie aseptisch waren und nicht zu stark bluteten.“ [24, S. 60]

Die örtliche Bevölkerung war an derartige Freiluftaktivitäten nicht gewöhnt und verständigte mindestens einmal die Gendarmerie, weil die turnenden Patienten für geisteskrank gehalten wurden [3, S. 135]. Solche Vorfälle bestärkten die Schlagintweits in ihren Plänen, ein eigenes Sanatoriumsgebäude mit abgezäuntem Park zu errichten. Dieses nahm ab 1910 Gestalt an und Oskar Schlagintweit übernahm die alleinige Leitung und die chirurgischen Operationen. Felix hingegen konzentrierte sich auf die konservative Therapie in seiner Münchner Praxis und transferierte nur besonderer Ruhe bedürftige chronisch Kranke nach Bad Brückenau.

Während sich Schlagintweit nach dem seiner Brückenauer Zeit ein Haus in der Münchener Heßstraße Maxvorstadt erwirbt, praktiziert er im Stadtzentrum in der noblen Maximilianstraße.

Die Verlagerung der operativen Eingriffe in den ländlichen fränkischen Kurort folgte wahrscheinlich der Logik, dass die Nachbehandlung mit den Wasserkuren vor Ort überwacht werden konnte und die Patienten bei Erkrankungen im Zusammenhang mit Syphilis oder Gonorrhoe weniger Gefahr liefen, sich gleich vor Ort neu zu infizieren. Das eigene Sanatoriumsgebäude gestattete die Kontrolle der Patienten. Der Komplex war vom Würzburger Baugeschäft Anton Eckert im Juli 1909 entworfen und den Behörden präsentiert worden.^10^

Da den Brüdern die Bearbeitungszeit zu lang erschien, beteiligten sie sich am bayerischen Volkssport des Schwarzbaus und ließen umgehend die Baugrube ausheben und mit der Aufmauerung des Kellers beginnen. Das Bezirksamt benötigte mehrere Monate, ehe man bemerkte, was auf dem Grundstück vor sich ging. Letztendlich rang sich das königliche Landratsamt im Mai 1910 zu der Feststellung durch, dass man die Baugenehmigung vom Juli des vorherigen Jahres rückwirkend erlasse und man der Vollendung des bereits weitgehend fertig gebauten Gebäudes freudig entgegen sehe.^11^ Oder anders formuliert: für eine mit den Spitzen der bayerischen Gesellschaft verkehrende bürgerliche Dynastie galten andere Maßstäbe als für gewöhnliche Untertanen. Die wenig später errichteten „Autohallen mit Salette-Aufbau“ wurden dann gleich ganz ohne Genehmigung errichtet. Auch in den folgenden Jahren gab es immer wieder Erweiterungen. Eine behördliche Genehmigung war erst im Jahre 1939 eingeholt worden, als Splitterschutzeinrichtungen vorgenommen wurden und die Zufahrtsstraße erweitert wurde.^12^ Zu dieser Zeit praktizierte der „bayerische Sanitätsrat, Spezialarzt für Nieren‑, Blasen‑, Harn- und Steinkranke“ Oskar Schlagintweit ganzjährig allein vor Ort [25, S. 30]. Sein Bruder Felix hatte sich bereits zu Beginn der 1930er-Jahre vollkommen aus dem Sanatoriumsbetrieb verabschiedet und sich auf die Praxis in München konzentriert. Felix behandelte vorrangig Männer, während Oskar sich offenbar zunehmend auf die „Erkrankungen der weiblichen Sexualorgane“ konzentrierte [26, S. 29].

## Künstler und Bohemien in München

Wie bereits bei seiner wissenschaftlichen Würdigung aufgeführt, war Felix Schlagintweit gesellschaftlich in die Münchener Künstlerszene eng eingebunden. Hierbei spielt der 1880 gegründete und im Münchner Kulturleben fest verankerte „Münchner Orchesterverein“ eine durchaus wichtige und prägende Rolle [127].

„Da gerade der O.V. die namhaftesten Münchner Künstler auf dekorativem Gebiet, Maler, Bildhauer, Architekten und Kunstgewerbler zu den Seinen zählte, so konnte er durch Verbindung von Musik und dekorativer Kunst Feste veranstalten, die weit über München hinaus berühmt wurden“ [3]

Schlagintweit, der Kontrabass spielte, freundet sich mit dem als Orchestervorstand fungierenden Architekten Emanuel von Seidl (1856–1919) und dessen Bruder Gabriel (1848–1913) vor dem ersten Weltkrieg an. Architekt Seidel war der wiederum mit Franz Stuck (1863–1928), Friedrich Kaulbach (1822–1903), Wilhelm Busch (1832–1908) und Franz von Lenbach (1836–1904) in der Künstlergesellschaft „Allotria“ verbunden. In engem Kontakt war Felix Schlagintweit außerdem mit den in München gesellschaftlich den Ton angebenden Familien von Pocci sowie von Riemerschmid-Zumbusch. Der Maler Ludwig von Zumbusch (1861–1927) vermittelte Felix Schlagintweit im Jahre 1905 den Kauf eines Hauses auf der idyllischen Halbinsel Urfahrn am Chiemsee, so dass der Arzt auch zum Kreis der gut situierten Münchner Landhausbesitzer zählte. Nach der Zerbombung seines Münchner Domizils im Jahr 1944 zog sich Felix Schlagintweit ganz nach Urfahren zurück (Abb. 14 und 15).

## Die Zeit nach 1930

**Figure 12:**
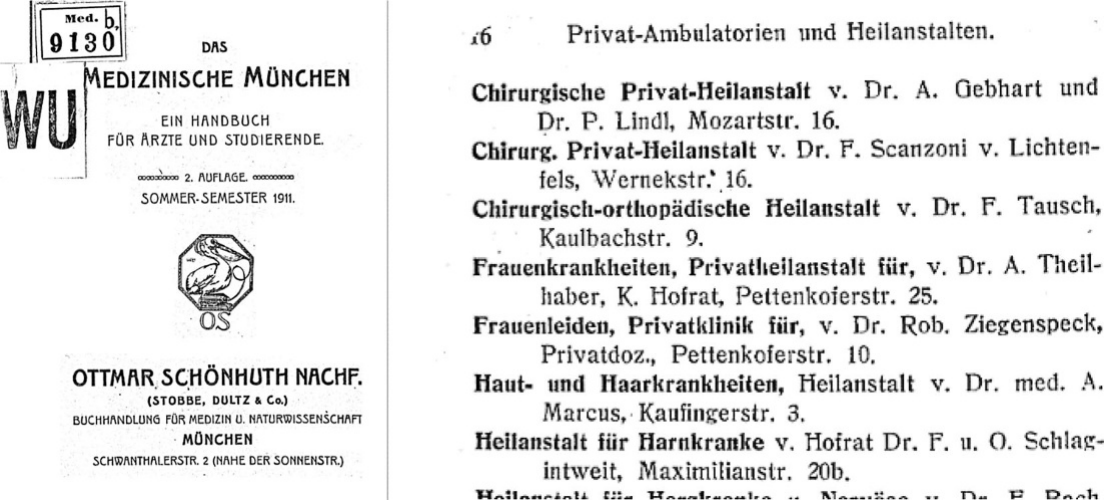

**Figure 13:**
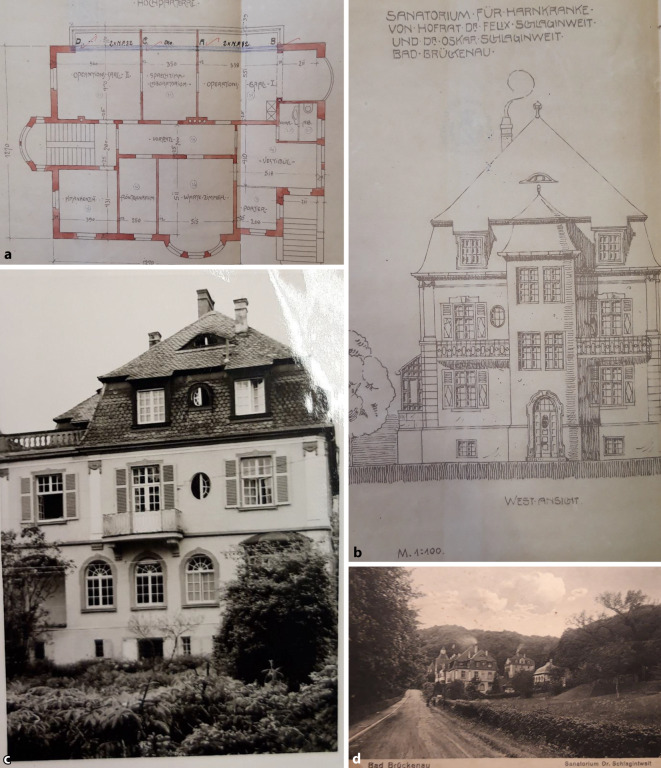

**Figure 14:**
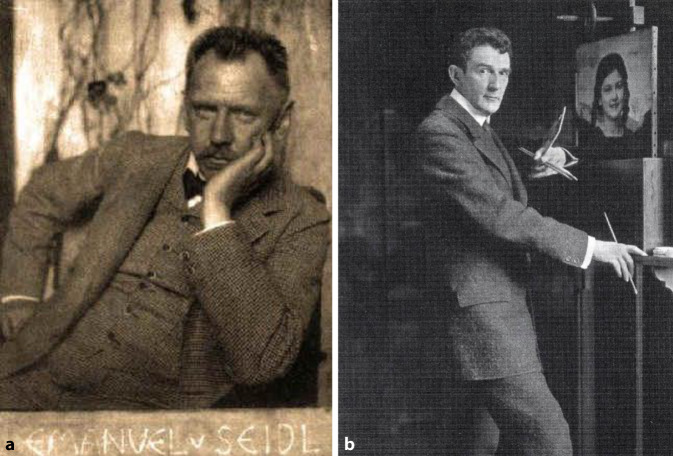

**Figure 15:**
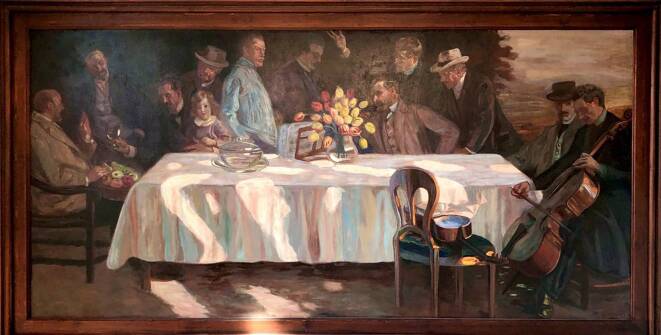

**Figure 16:**
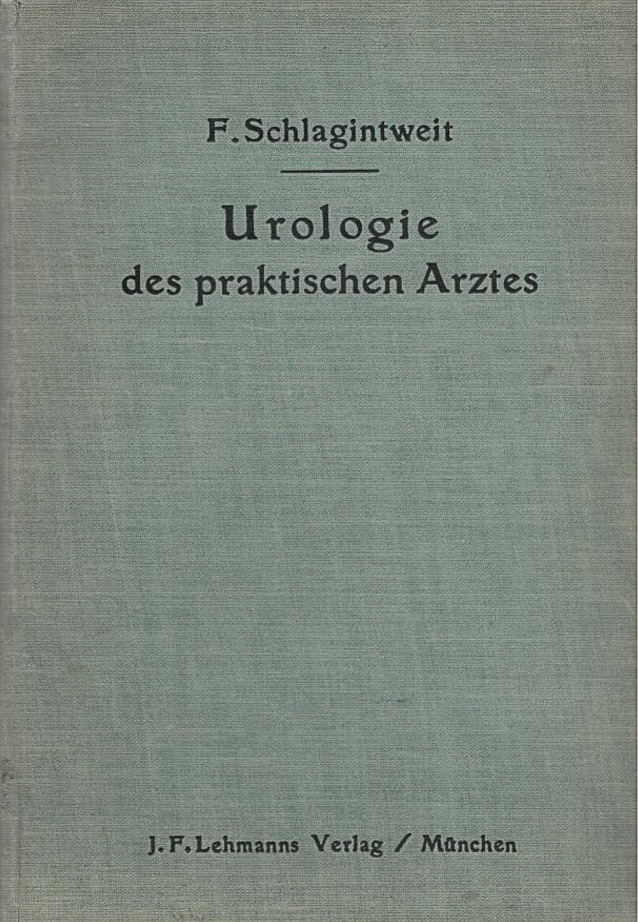

**Figure 17:**
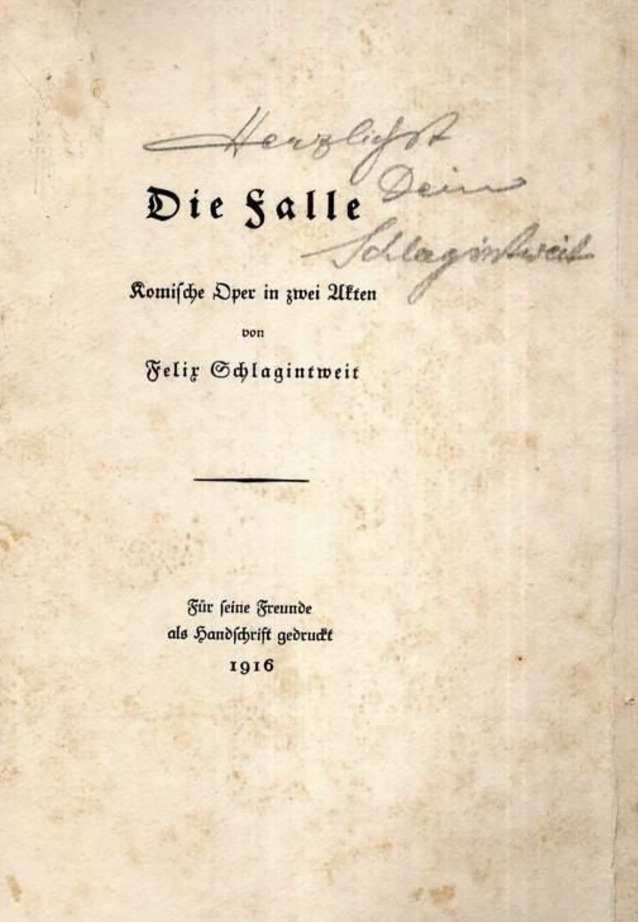

**Figure 18:**
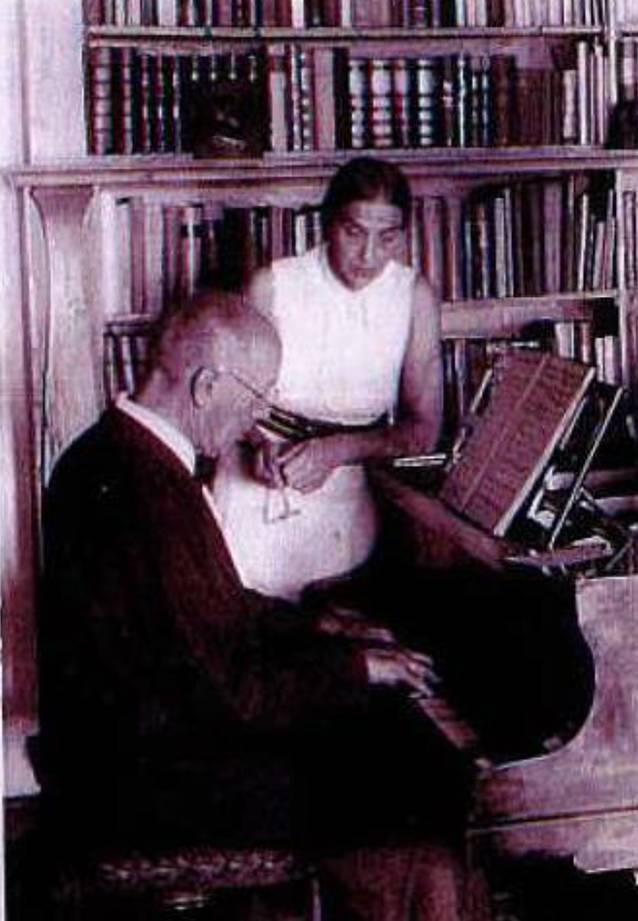

Felix Schlagintweit war aufgrund seiner balneologischen Ausrichtung ein besonderer Anhänger konservativer, minimal-invasiver Therapien und sie fanden eine zahlungskräftige Anhängerschaft. Doch die Ärzteschaft sah sich in den 1920er-Jahren mit neuen Herausforderungen konfrontiert. Die Kaufkraftverluste durch Kriegsniederlage und Inflation, die sich immer stärker formierende Fraktion der Laienheilkundigen und Homöopathen sowie die Arbeitsbedingungen in einem allumfassenden Sozialstaat verlangten nach schnellen, praktikablen Lösungen für eine große Zahl an Patienten. Diese Veränderungen machten auch vor der Urologie nicht Halt. Ihre Fachvertreter sahen in einer Spezialisierung auf operative Methoden eine gute Lösung, was Schlagintweit wenig schmeichelhaft als „urologischen Dilettantismus“ abkanzelte [28]. Seiner Ansicht nach gehe so jede Kompetenz zur Psychosomatik verloren und zugleich bestünde die Gefahr, dass unerfahrene Ärzte mit urologischen Techniken hantierten, was dem Ansehen des Faches schade. Dass der Schlüssel zu einer erfolgreichen Therapie in der sorgsamen und individuellen Diagnose lag, hatte er bereits 1921 in einer ausdrücklich an Fachfremde gerichteten Einführung herausgestellt [36]. Hier empfahl er auch jene direkte Frage für das Arzt-Patient-Gespräch, die den Titel des vorliegenden Aufsatzes beinhaltet (Abb. 12, 13, 14, 15, 16, 17, 18).

Nach 1933 betonte er, basierend auf einem Patientenmaterial von mehr als 3000 Fällen, dass konservative Therapien bei der Prostatahypertrophie der operativen Methode nicht unterlegen seien [29]. Auch die diagnostischen Pannen bei Nierensteinen ließ er nicht unerwähnt [30]. Er nahm an, dass Katheterisieren auf Dauer eine Operation überflüssig machen könne [6, S. 55]. Diese bedeutsamen katamnetischen Studien erschienen bezeichnenderweise nicht mehr in der bei Georg Thieme in Leipzig herausgegebenen fachprägenden *Zeitschrift für Urologie* sondern in der nicht ganz so angesehenen *Zeitschrift für ärztliche Fortbildung*, in deren Herausgebergremium Urologen auch keine Netzwerke entwickelt hatten. Schlagintweit verweigerte sich auch der Neuorganisation des Fachs in Gestalt der „Gesellschaft Reichsdeutscher Urologen“ [31, S. 23–40]. Es gibt keine Hinweise, dass er sich an der eugenischen Praxis beteiligt hätte. Stattdessen wählte er den Gang ins innere Exil. Er verfasste weiterhin Theaterstücke, schrieb Gedichte und veröffentlichte 1935 eine medizinhistorische Abhandlung über Krankheit und Tod des französischen Kaisers Napoleon III (1808–1873, reg. 1852–1870) [32].

Der an Blasensteinen leidende Kaiser Napoleon III wird von ihm als ein verhinderter Sozialist auf dem letzten französischen Kaiserthron portraitiert. Reichskanzler Fürst Bismarck, Kaiserin Eugenie und ihr Hofstaat, Kaiser Maximilian von Mexiko, Viktor Emanuel II. von Italien werden in dieser breit angelegten Skizze europäischer Geschichte eingebunden. Mit ihrem Untertitel ‚Menschliches – Allzumenschliches aus dem zweiten Kaiserreich‘ gibt der Text den Erzählansatz Schlagintweits programmatisch vor, der „die geschichtliche Wahrheit in Anekdoten“ und „die wirklichen Menschen von damals“ zu schildern versuchte.

In seinem postum veröffentlichten, aber bereits in den 1930er-Jahren verfassten Roman „Don Juans Hochzeitsreise“ erteilte er „Politik und Weltanschauung“ eine klare Absage [33].

Der autobiografisch anmutende Roman, beschreibt eine Dreiecksbeziehung zwischen einer Frau und zwei Männern. Der einst lebenslustige und schriftstellerisch ambitionierte Arzt Lutz ist durch den Zweiten Weltkrieg zum Invaliden geworden, sein Konkurrent Prof. Will kehrt von der Ostfront nicht zurück. Lutz begeht auf dem stürmischen Chiemsee einen Selbstmordversuch. Lisa rettet ihn, und die beiden finden in tiefer Liebe wieder zueinander.

Nachdem sein Wohnhaus und Praxis in München im Bombenkrieg abgebrannt waren, zog er sich mit der zweiten Ehefrau in sein Sommerhaus am Chiemsee zurück. Hier vollendete er seine Memoiren, in denen er mit Kritik an der Kollegenschaft nicht sparte und ganz nebenbei noch den Wert von Hormonen für die urologische Behandlung herausstellte [6, S. 56f.]. Er starb am 17. Mai 1950 an den Folgen einer Mesenterialvenenthrombose und wurde auf eigenen Wunsch hin auf dem kleinen Friedhof der Fraueninsel bestattet. Ein Jahr zuvor hatte die wiedergegründete Deutsche Gesellschaft für Urologie (DGU) ihn zum Ehrenmitglied ernannt [14].

## Nemesis

Zum Zeitpunkt seines Todes am 18. Mai 1950^13^ [14] war Felix Schlagintweits Name bereits in den Annalen der deutschsprachigen Urologie verblasst. Die Integration der Antibiotika in die urologische Praxis ab den 1950er-Jahren ließ den Wert anderer konservativer Therapien zunehmend schwinden. Zeitgenössische Kollegen, die seine Memoiren zur Hand nahmen, dürften von der Lektüre wenig angetan gewesen sein. Erst im Jahre 1978 wurde Felix Schlagintweit dem Vergessen ein wenig entrissen, weil der Münchner Lehrstuhlinhaber für Geschichte der Medizin Heinz Goerke (1917–2014) durch seinen Doktoranden Klaus Voß eine Dissertation über Schlagintweit anfertigen ließ [6]. Im Fach Urologie setzte die Erinnerung erst nach der Jahrtausendwende wieder ein. Seinen fachinternen Gegnern erging es im Übrigen nicht besser als ihm. Ihre Vorgehensweisen wurden spätestens ab den 1960er-Jahren durch die Einführung von antibakterieller Chemotherapie und Laserbehandlungen aus dem Diskurs verdrängt [34, S. 241–246, 242, 35, S. 223–227, 223]. Die Verwicklungen der begeisterten Anhänger des Skalpells in die Verbrechen des Nationalsozialismus trugen ebenfalls dazu bei, sie aus der Ahnenhalle der Urologie zu tilgen. Hier ist nun auch Platz für Personen wie die Felix Schlagintweit und Bruder Oskar. Auch sie waren „Entdecker“, wie ihre einst berühmten Vorgänger.

## Zusammenfassung – Fazit für die Praxis

Der Lebensweg Felix Schlagintweits zeigt fokussiert die Möglichkeiten auf, die sich jungen Ärzten im Großstadtbereich boten, die neue Spezialdisziplin der Urologie sowohl akademisch wie praktisch zu vertreten.

Hierbei waren Auslandaufenthalte an wesentlichen Zentren wie Paris oder London vor Einführung der Facharztprüfung 1924 sinnvoll und in Fachkreisen anerkannt zum Wissenserwerb und für die Urologie fachprägend für diese Generation von Urologen.

Während viele Krankenhausmediziner versuchten, eine eigenständige Stellung neben anderen operativen Fächern wie Chirurgie oder Gynäkologie zu erhalten, war die operative Tätigkeit der Urologen der venerologisch-endoskopischen Richtung auf ein minimal-invasives Vorgehen (exemplarisch blinde Blasensteinlithotripsie), das sie in eigener Praxis oder Privat- oder Kurklinik gut ausführen konnten, ausgelegt.

München gehörte neben Wien zu den Orten, die sich dem Diskurs um Habilitationen im neuen Fach im Gegensatz zu Berlin nicht verschlossen, auch wenn – aus bisher noch unbekannten Gründen – im Jahre 1912 Schlagintweit im Gegensatz zu Ludwig Kielleuthner noch nicht erfolgreich war.

## Footnotes

1 Bamberger Volksblatt 19.04.1895 23 Jahrgang Nr 89, Standesamtliches, Verlobungsanzeige 14. August 1893 Augsburger Abendzeitung Nr 255, S. 3

2 Münchner neueste Nachrichten. 1898 Jg. 51, 4. Juni 1 S. 3

3 Mitgliederliste 1907 Mitgliederverzeichnis DGU in Verhandlungen der Deutschen Gesellschaft für Urologie. Georg Thieme, Leipzig, S. 15

4 Universitätsarchiv München, W‑I-88, Band 5, Schlagintweit, Bl. 176 f.

5 Universitätsarchiv München, W‑I-88, Band 5, Schlagintweit, Bl. 174

6 Universitätsarchiv München, Kiehlleutner N I 89 Band 5, E II 78

7 Personalakte Ludwig Kielleuthner Universitätsarchiv München

8 Der Lehmanns Verlag wurde 1890 von Julius Friedrich Lehmann (1864–1935) gegründet. Er entwickelte sich zu einem wichtigen deutschen Fachverlag für Medizin und publizierte seit dem Ersten Weltkrieg zunehmend auch rassenkundliche sowie wehrwissenschaftliche und rassenhygienische Schriften. Seit dem Jahr 1929 unterstützte der Verlag offen die NSDAP. Nach dem Zweiten Weltkrieg 1950 als medizinischer Verlag wiederbegründet, publizierte er weiterhin auch wehrwissenschaftliche Publikationen. Der Verlag wurde 1979 aufgelöst (Heidler M 2006 Historisches Lexikon Bayerns online: https://www.historisches-lexikon-bayerns.de/Lexikon/J.F._Lehmanns_Verlag zugegriffen 23.03.2023).

9 Letzter von der Königlich Bayerischen Regierung ernannter Geheimer Hofrat war 1918 der Chirurg Ferdinand Sauerbruch gewesen. Ferdinand Sauerbruch, Hans Rudolf Berndorff: Das war mein Leben. Kindler & Schiermeyer, Bad Wörishofen 1951 S. 240

10 Staatsarchiv Würzburg, Landbauamt Bad Kissingen 1314, Entwurf Sanatorium für Harnkranke

11 Staatsarchiv Würzburg, Landratsamt Bad Brückenau 2695, Brief der Gebrüder Schlagintweit an das Landratsamt vom 10.08.1909, Antwortschreiben 13.08.1909

12 Staatsarchiv Würzburg, Landbauamt Bad Kissingen 1314, Bauanweisung vom 17.08.1939

13 Standesamt Breitbrunn Sterbebuch 18. Mai 1950 Nr 6 mail Notburga Bauer VG Breitbrunn 03.11.2022
